# Editorial: Hypertension and Chronic Kidney Injury or Failure, Volume II

**DOI:** 10.3389/fphys.2021.824971

**Published:** 2022-01-07

**Authors:** Zhengrong Guan, Suttira Intapad, Oleg Palygin, Jennifer C. Sullivan

**Affiliations:** ^1^Division of Nephrology, Department of Medicine, University of Alabama at Birmingham, Birmingham, AL, United States; ^2^Department of Pharmacology, School of Medicine, Tulane University, New Orleans, LA, United States; ^3^Division of Nephrology, Department of Medicine, Medical University of South Carolina, Charleston, SC, United States; ^4^Department of Physiology, Medical College of Georgia, Augusta University, Augusta, GA, United States

**Keywords:** Ang II, hypoxia, Wnt/β-catenin, hypoxia-inducible factor (HIF), vascular endothelial growth factor (VEGF), polycystic kidney disease (PKD)

## Introduction

The prevalence of chronic kidney disease (CKD) has dramatically increased in the past two decades and has become a significant public health problem globally. Epidemiological studies indicate that over 37 million American adults suffer from CKD, with most of them are not aware that they have renal disease (Murray et al., 2021). This fact dramatically increases the risk of irreversible damage to the kidneys and end-stage renal disease (ESRD). Kidney diseases have risen from the world's 27th leading cause of death in 1990 to the 8th in 2019 (Heron, 2021). Hypertension is one of the common causes of CKD and present in ~19.5% of CKD patients. Therefore, a better understanding of the mechanisms underlying hypertension-associated kidney injury and CKD progression is a major public health goal. We published five articles with two reviews and three original basic research articles in the second collection of Hypertension and Chronic Kidney Injury or Failure. Those original studies and review articles provide novel mechanisms and new insights for the prevention of CKD progression.

## Intratubular, Intracellular, and Mitochondrial Angiotensin II/AT1 Receptor/NHE3 Signaling in Hypertension and Kidney Injury

The main purpose of this review article by Li et al. is to focus on refractory angiotensin II (Ang II)-dependent hypertension through elucidation of an AT_1_ (AT_1a_) receptor-dependent mechanism and intratubular, intracellular, and mitochondrial system interactions. The authors summarized recent progress regarding the role of circulating Ang II in proximal tubules. They conclude that intratubular Ang II interacts with the proximal tubule cells to further induce blood pressure responses. Moreover, overexpression of Ang II fusion protein in mitochondria led to the development of hypertension. Finally, the deletion of AT_1_ (AT_1a_) or Na^+^/H^+^ exchanger 3 in proximal tubules reduced basal blood pressure and Ang II-induced hypertension. Ang II is reported to be degraded through the lysosome degradation pathway. However, the authors found that it might bypass the degradation pathway and subsequently be transported to mitochondria and other cellular elements, activating mitochondrial and nuclear AT_1a_ receptors. Together with other evidence, the authors propose that the proximal tubule intratubular, intracellular, and mitochondrial Ang II system plays an essential role in hypertension and renal injury.

## Molecular Mechanisms of Polycystic Kidney Diseases

Polycystic kidney disease (PKD) is one of the most commonly inherited diseases and is characterized by the continuous increase of kidney size due to the development of enlarging cysts in kidneys, eventually resulting in develops chronic kidney injury and ESRD. PKD is defined into two forms: the autosomal dominant PKD (ADPKD) and the autosomal recessive PKD (ARPKD). This review article by Vasileva et al. discusses the recent advances and insights on the molecular mechanisms and pathogenesis of PKD, with a stronger focus on ADPKD. The authors also provide details on the mechanisms and side effects of Tolvaptan, the only medication FDA approved for ADPKD (not ARPKD) treatment. In addition, the importance of renin-angiotensin-aldosterone system blockade and management of hypertension, one of the common complications of ADPKD, is discussed. The manuscript also touches on the possible involvement of hormones, growth factors, mitochondria abnormalities, and inflammation in the cyst progression. Finally, the authors discussed recent findings on different dietary interventions that could profoundly affect PKD progression.

## Prolonged Ang II Exposure on Oxidative and Endoplasmic Reticulum Stress, and Na^+^ Transporters

Elevations in circulating Ang II plays a vital role in the development of hypertension and CKD. The study by Lins et al. aimed to investigate the contribution of prolonged Ang II exposure to oxidative and endoplasmic reticulum (ER) stress in rat kidneys and its long-term impact on tubular Na^+^ transport. The paper discusses the impact of late pharmacological interventions with an AT_1_ receptor antagonist, Losartan (2 weeks after a 4-weeks Ang II infusion), on the adverse effects of the extended Ang II exposure on kidney injury. The authors provided compelling evidence, demonstrating that activation of AT_1_ receptors promoted the transcription of different NADPH oxidase subunits as well as ER stress. The study also highlights the therapeutic potential of AT_1_ blockers on Na^+^ reabsorption via NHE3, ENaCβ, NKCC2, and NCC transporters. In summary, this original study suggests the importance of pharmacological blockade of Ang II/AT_1_ receptor pathway to reduce oxidative and ER stress and the deleterious effects of pro-inflammatory and pro-fibrotic processes, which ultimately can accelerate chronic kidney injury progression.

## Wnt/β-Catenin Signaling in AKI and the CKD Transition

Acute kidney injury (AKI) is a common clinical complication and is now recognized as a high-risk factor in CKD progression. Despite intensive research, the underlying mechanisms of AKI to CKD transition is still not well-understood. The Wnt/β-catenin signaling pathway is well-recognized in the field of kidney diseases (Huffstater et al., 2020); however, the exact mechanisms of its activation and inhibition remain elusive in the injured kidney. The current report by Hong et al. addresses the role of Wnt/β-catenin signaling in AKI prevention and the transition to CKD using an ischemia-reperfusion (IR)-induced AKI (IR-AKI) mouse model. The authors found that injection of exogenous Wnt1 expression vector prior to IR impeded the progression of AKI to CKD. Intriguingly, activation of exogenous Wnt1 protected from tubular necrosis and inflammation in mouse IR-AKI kidneys and prevented kidney fibrosis in IR-CKD mouse kidneys. However, the inhibition of Wnt/β-catenin signaling pathway before IR had no effect. Consistent with their previous report (Xiao et al., 2016), the authors concluded that activation of Wnt/β-catenin signaling protects kidneys against AKI, whereas sustained elevation of this pathway could accelerate the progression from AKI to CKD. Therefore, timing and duration of treatment with exogenous Wnt1 are critical in determining the fates of kidney function after IR-AKI. The study suggests that targeting the Wnt/β-catenin pathway could be a potential therapeutic approach for preventing AKI to CKD progression.

## Hypoxia-HIF-VEGF Signaling in Kidney Injury

Hypoxia has been implicated in contributing to the development of renal interstitial fibrosis and subsequently may lead to irreversible CKD. Kidney hypoxia triggers inflammatory cascades and alters hypoxia-inducible factor (HIF) pathways by upregulating or downregulating downstream target gene expression. The current study by Xu et al. determined how hypoxia impacted the HIF-vascular endothelial growth factor (VEGF) signaling pathway in neovascularization in a rat model of hypoxia. The authors found that both HIF-1a and VEGF expression were elevated in kidneys of hypoxic rats that received mild hypoxia (10% O_2_) or severe hypoxia (7% O_2_) over 7 or 14 days. Furthermore, the mild hypoxic group showed subtle renal injury after 7 days of exposure to hypoxia. However, continuous exposure to hypoxia eliminated the kidney injury along with significant increases in VEGF protein expression and mature blood vessel density. Interestingly, the renal injury was aggravated under severely hypoxic conditions, and inflammatory factors (IL-6, IL-10, and TNF-α) were markedly increased over time while the mature blood vessel density was reduced compared to the sham control and mild hypoxic groups. Overall, this study highlights a pivotal role of the HIF-VEGF signaling pathway for angiogenesis regulation under the condition of hypoxia. Mild hypoxia could stimulate the proliferation and migration of vascular endothelial cells and promote neovascularization, allowing kidneys to adapt a protection mechanism for self-repair. However, severe hypoxia can trigger kidney damage by stimulating inflammatory cascades and renal fibrosis, ultimately causing irreversible pathological changes.

## Conclusion

The Research Topic, Hypertension and Chronic Kidney Injury or Failure, highlights novel mechanisms that contribute to acute and chronic kidney injury induced by either hypoxia, ischemic insult, or prolonged Ang II exposure. The original research articles provide compelling evidence demonstrating that targeting specific signaling pathways may be necessary for successful therapeutic approaches to prevent AKI and CKD progression. The review articles summarize the most promising avenues of research in polycystic kidney diseases and blood pressure regulation driven by the intratubular and mitochondrial signaling pathways.

## Author Contributions

ZG drafted the manuscript. ZG, SI, OP, and JS edited and revised the manuscript. All authors contributed to the article and approved the final version of the manuscript for the submission.

## Funding

This work was supported by the NIH NIDDK DK106500 (ZG), NIGMS 5P20GM109036 (SI), and DK126720 (OP).

## Conflict of Interest

The authors declare that the research was conducted in the absence of any commercial or financial relationships that could be construed as a potential conflict of interest.

## Publisher's Note

All claims expressed in this article are solely those of the authors and do not necessarily represent those of their affiliated organizations, or those of the publisher, the editors and the reviewers. Any product that may be evaluated in this article, or claim that may be made by its manufacturer, is not guaranteed or endorsed by the publisher.
